# Supplementary material for: Viral-bacterial co-infections screen in vitro reveals molecular processes affecting pathogen proliferation and host cell viability
Source: Nat Commun. 2024 Oct 4;15:8595. doi: 10.1038/s41467-024-52905-2 (PMC11452664; doi:10.1038/s41467-024-52905-2)
Supplement: Supplementary file 1 — Supplementary Information [file 41467_2024_52905_MOESM1_ESM.pdf]

Supplementary Figures

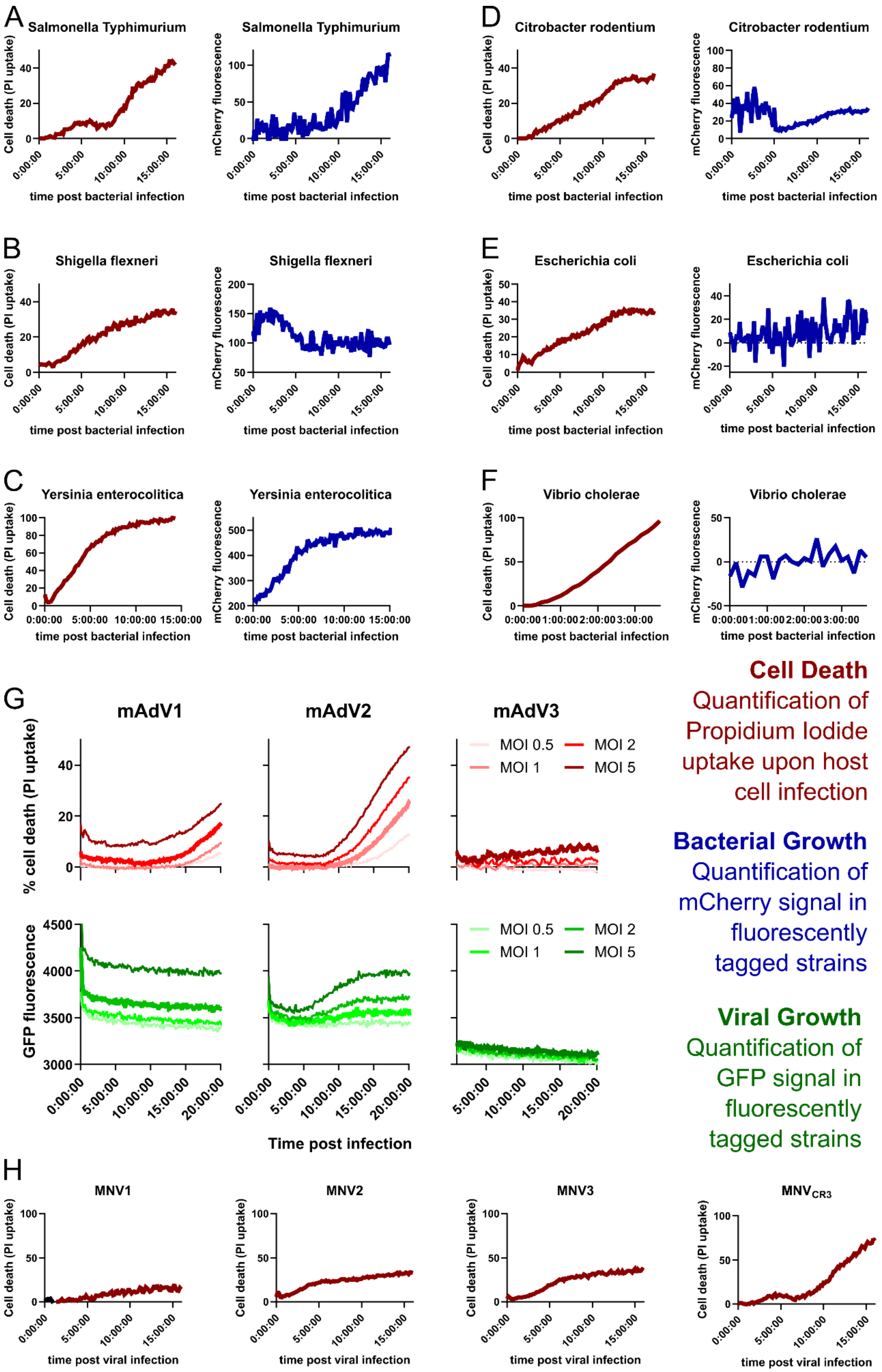

Figure S1: Single-pathogen infection dynamics of the strains used in the co-infection study. A-F) Host cell death (red curves, measured by quantification of propidium iodide uptake over time) and bacterial growth (blue curves, measured by quantification of mCherry signal) related to the six bacterial pathogens, all used at an MOI of 50. Means of technical triplicates or duplicates are depicted in the curves, representative plots are shown. G) Viral titration in the single-infection setting. Different MOIs (as indicated by shade) were used in infection and viral growth (green curves, quantified by GFP-signal of fluorescently tagged strains), as well as host cell death (as in panels A-F) was quantified over time. The means of technical quadruplicates of one representative biological replicate are shown. H) Cell death quantification (as in panels A-F) for single infection with MNV-strains used in the study. Representative curves (means of technical triplicates) are shown, and viral infection was performed at the MOI depicted in Table 1 of the Methods section: MNV1: MOI=1, MNV2: MOI=1, MNV3: MOI=3, MNV_{CR3}: MOI=0.25.

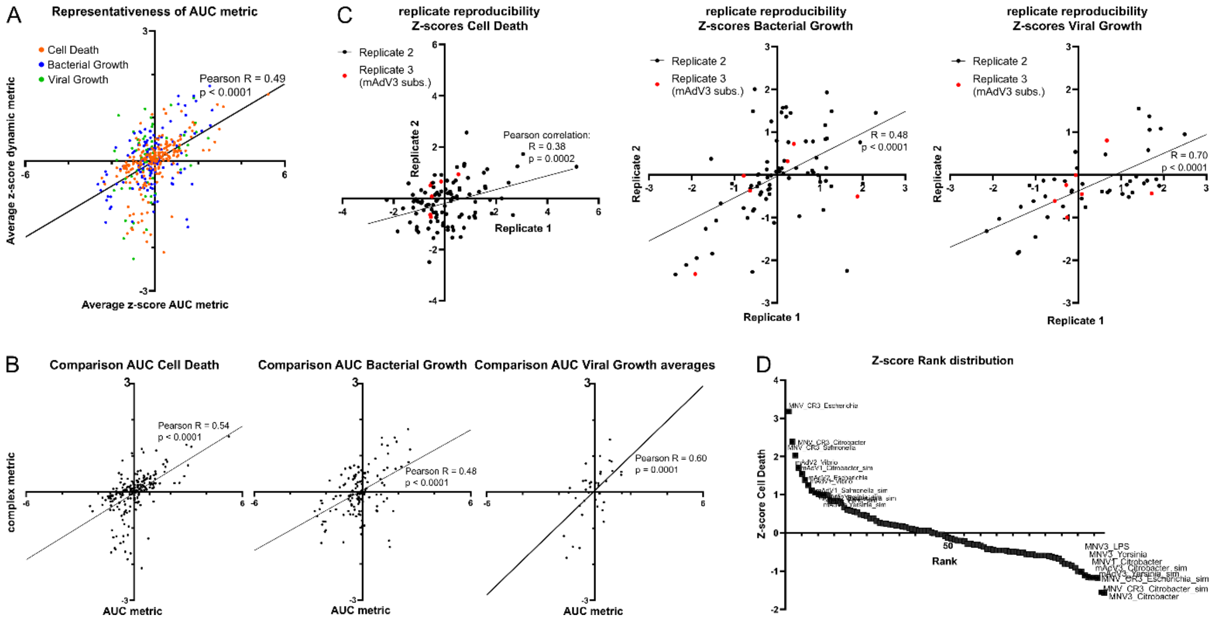

Figure S2: Quality control of the pairwise co-infection screen. A) Scatterplot depicting the correlation between the AUC-metric-based z-score (x-axis) and the average of the dynamic metrics (onset of growth / death, half-point, gradient, maximum growth / death) z-normalized (y-axis). Each dot represents an interaction pair in a specific readout (cell death, bacterial growth or viral growth, as color-coded). Pearson correlation, and a linear approximation are indicated. The significance of deviation of the slope from zero was tested (two-sided test) and is provided by the p-value shown in each plot. B) As panel A, but separate for each readout. C) Correlations of the two biological replicates for the z-scores (AUC-based) in each readout, as indicated. Each dot represents an interaction pair. For mAdV3 subsequent infection, a third biological replicate was conducted; the interactions stemming from this are indicated in red. Linear approximation, Pearson correlation and assessment of whether the slope is significantly different from zero (two-sided test) are indicated. D) Rank distribution of z-scores (host cell death, AUC-based) for each interaction. Interactions with an absolute z-score larger than 1 are indicated by label.

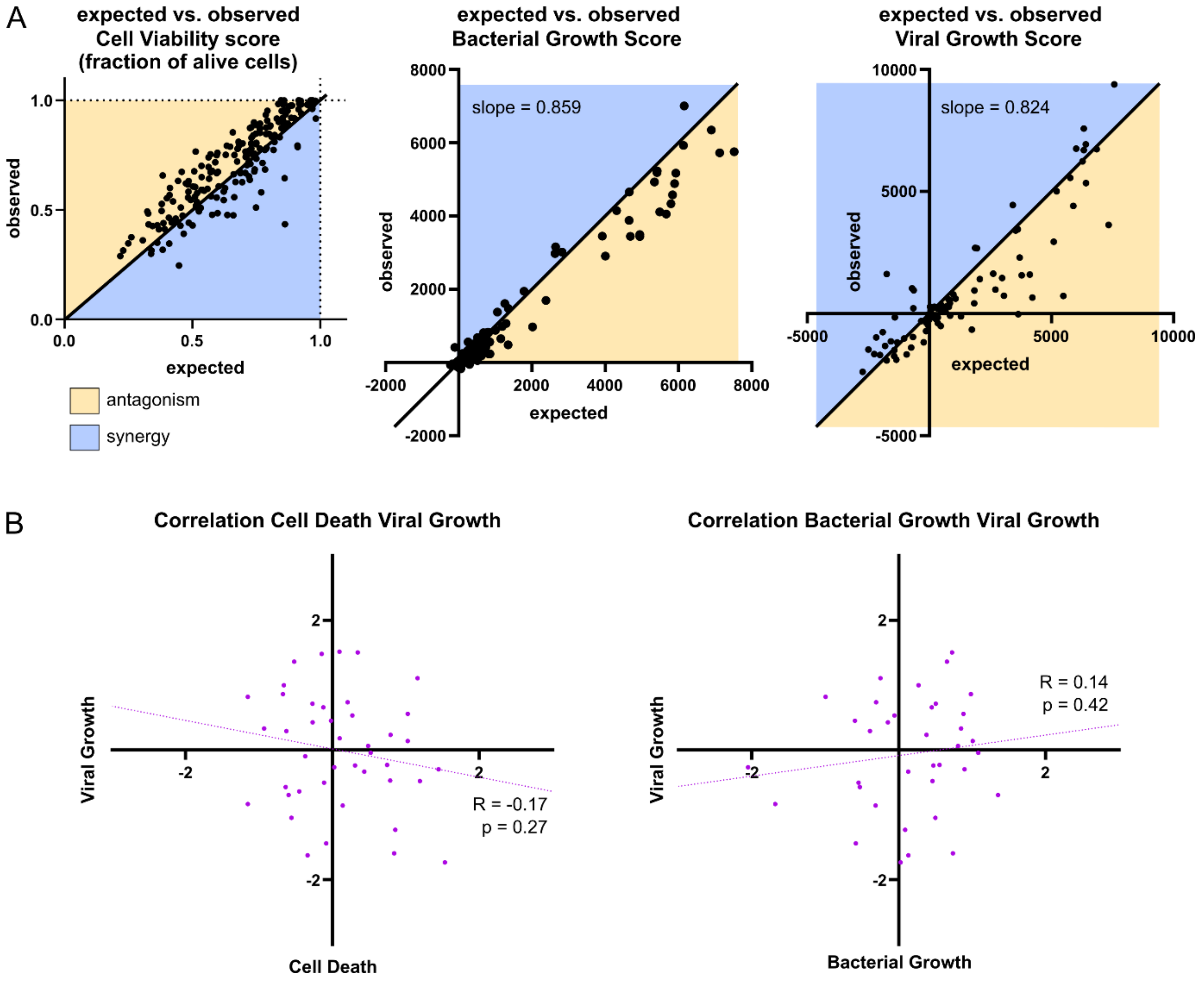

Figure S3: General trends observed in the pairwise screen. A) Scatterplots of expected and observed values (non-z-transformed Bliss scores) for the three assessed readouts as indicated. The line depicts the bisectrix and the area in the graph for synergies (blue) and antagonisms (orange) are indicated. Each dot represents one interaction pair. For the second and third panel, the slope of a linear correlation is indicated (the linear approximation itself is not depicted for reasons of readability) B) Correlation of z-scores as indicated, Pearson R and linear approximation, as well as whether the slope of said approximation is significantly non-zero (two-sided test) are indicated. Each point represents a pathogen pair, as in Fig. 2D.

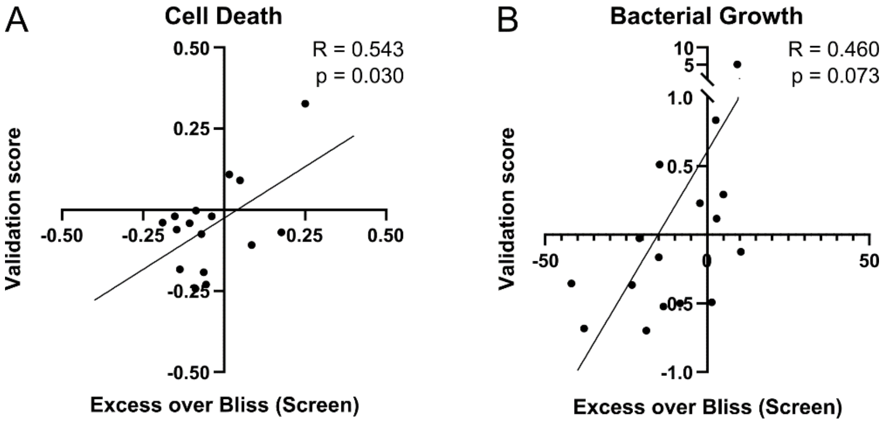

Figure S4: Correlations of the validation and the screening data. A) Scatterplot of interaction pairs selected for validation with respect to Cell Death. The x-axis depicts the Bliss Score obtained in the screen, based on PI uptake, while the y-axis represents the result of the validation, which was obtained through measurement of LDH-release for each pathogen-pair that was assessed individually. Pearson R and significance of deviation of the slope from zero (two sided test) are indicated. Each dot represents one tested interaction (N=16). B) As in A, but for the validation of Bacterial Growth. The x-axis thus represents the score obtained in the screening, based on fluorescence measurement (mCherry), and the y-axis depicts the validation based on CFU counting.

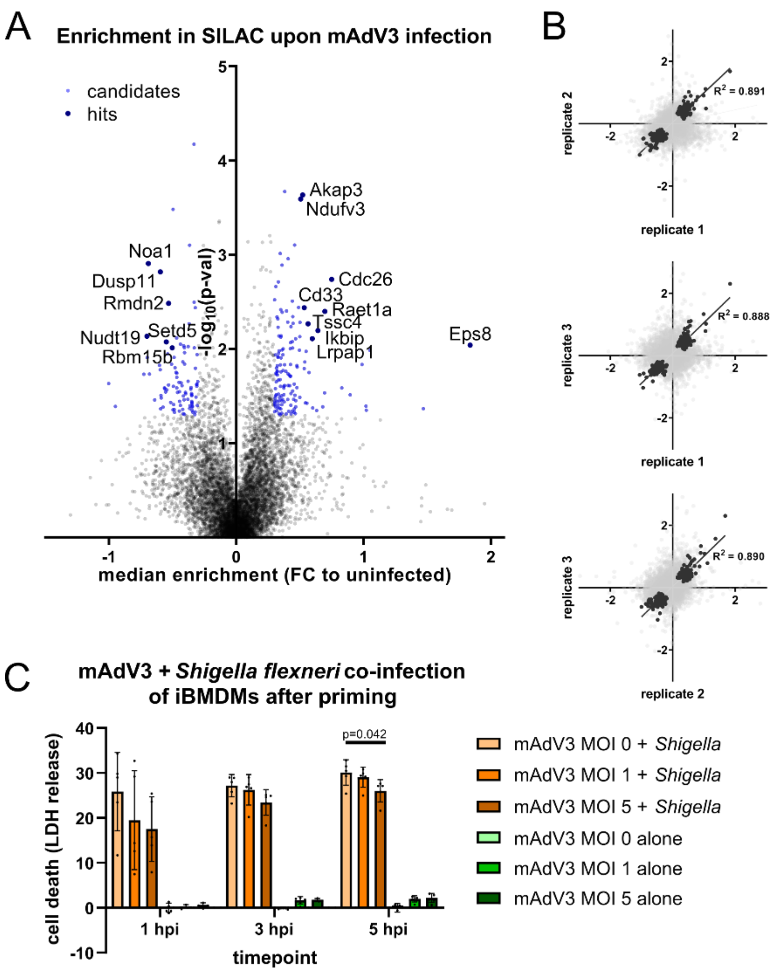

Figure S5: Host proteome alterations upon mAdV3 infection and impact on secondary bacterial infection. A) Volcano plot of SILAC enrichment (x-axis: median logarithmic fold change of three biological replicates, y-axis: negative logarithmic p-value, not corrected for multiple testing). Hits (absolute log(FC) > 0.5, p-value < 0.01) and candidates (absolute log(FC) > 0.3, p-value < 0.05) are indicated by name. Full results are available in Table S2 B) Pairwise correlations of the three replicates (log(FC) as indicated. Gray dots: All proteins, black dots: candidates, as defined in panel A. The line and R^2 value are based on the proteins classified as candidates. C) Cell death (measured by LDH release) assessment of mAdV3-*Shigella* co-infection over time. MOIs and timepoints are indicated. iBMDMs were primed with IFNγ for at least 6h and infected with the indicated MOI of mAdV3. After overnight infection, cells were infected for the indicated time with *Shigella* at an MOI of 50. Mean and standard deviation of five replicate wells are given. P-value was calculated using two-sided, unpaired Student's T-test with Welsh correction.

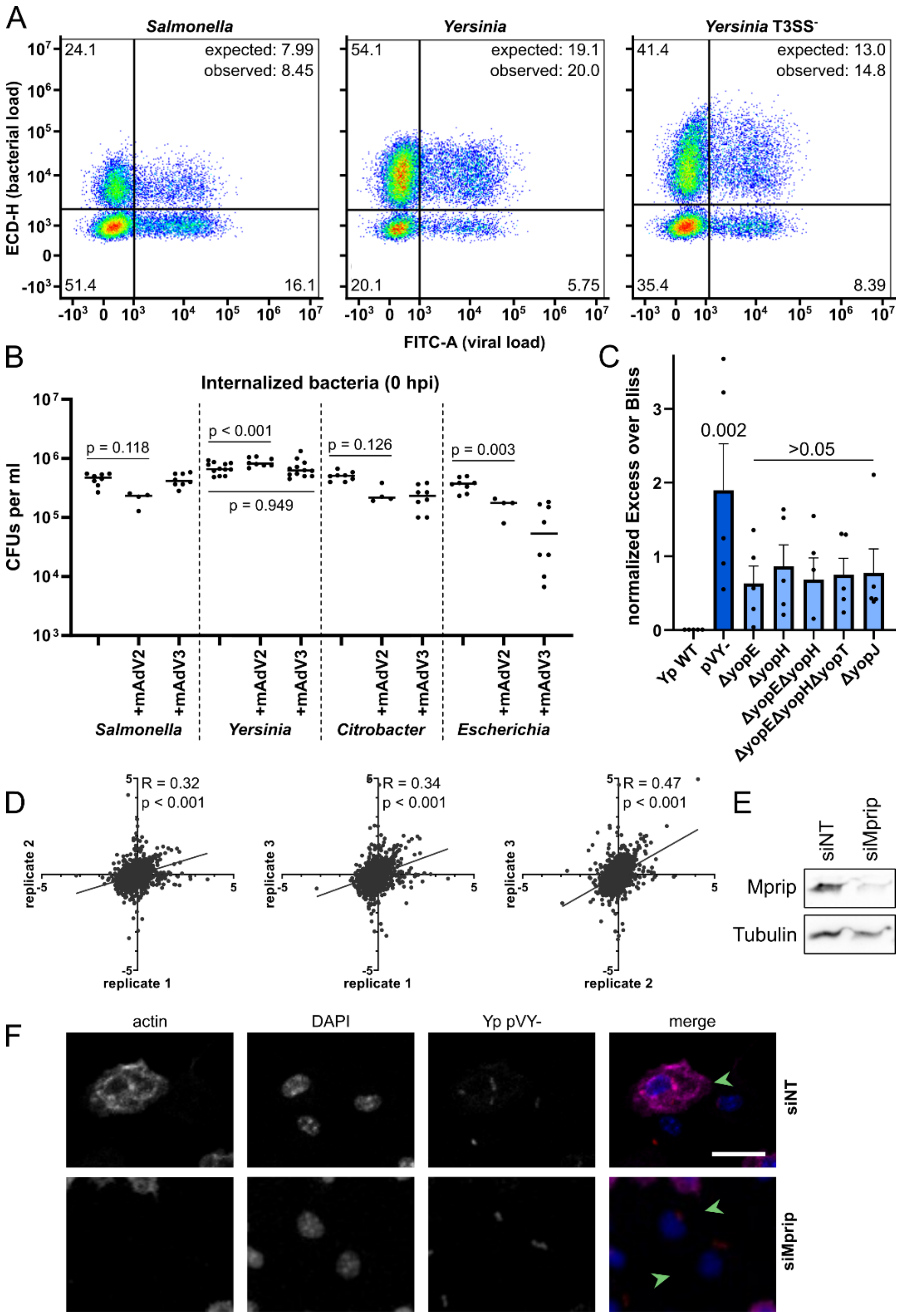

Figure S6: Increased host-cell uptake of *Yersinia* after mAdV2-infection and involvement of host and bacterial proteins at the interface. A) Representative scatterplots of FITC-signal (viral load) and ECD-signal (bacterial load) for co-infected cell-population. For each quadrant (uninfected, singly infected and doubly infected BMDMs), the fraction of all live cells are indicated. The expected fraction of co-infected cells was calculated by multiplying the fraction of virally and bacterially infected cells. B) CFU counting after invasion (time point 0hpi) for different bacterial pathogens after infection with mAdV2 or mAdV3. Each point represents a quantification across biological triplicates, p-values indicate unpaired comparisons by two-sided Student's T-Test using Welsh correction. C) Increase in observed co-infected cells for a panel of *Yersinia* mutants, normalized to WT *Yersinia pseudotuberculosis* during infection (MOI 5, time point 0hpi). Mean and standard deviation are indicated, this data is based on biological triplicate. P-values indicate significance based on one-way ANOVA, comparing all

mutants to the wildtype. D) Pairwise replicate correlation for the SILAC data displayed in Fig. 5C (logarithmic fold-change). Each dot represents a protein and Pearson R, alongside a linear approximation, including significance of whether the slope is non-zero (two-sided test) across all proteins are given. E) Knockdown validation of Mprip by siRNA through Western Blot, 48h after transfection of BMDMs. Tubulin is shown as loading control. Uncropped, original image, as well as additional replicates can be found in Source Data File. F) Representative microscopy images (crop from a field of view acquired with 20x magnification) used for the quantification in Fig. 5F,G. Actin, DAPI, bacterial signal (mCherry) and merge of virally infected cells (green arrows) are shown. To enhance visibility, grayscale images are shown, original images can be found at https://data.mendeley.com/datasets/thjzhzdpvc/1, as well as Source Data File. Scale bar: 20μm.
