# Supplementary material for: Viral-bacterial co-infections screen in vitro reveals molecular processes affecting pathogen proliferation and host cell viability
Source: Nat Commun. 2024 Oct 4;15:8595. doi: 10.1038/s41467-024-52905-2 (PMC11452664; doi:10.1038/s41467-024-52905-2)
Supplement: Supplementary file 3 — Description of Additional Supplementary Files [file 41467_2024_52905_MOESM3_ESM.pdf]

Description of Additional Supplementary Files

Supplementary Data 1:
Dynamic metrics z-scores: Z-transformed interaction scores based on the dynamic calculation (as described in the methods section). This data is the direct source for figure panel 1B
AUC metric EOB: Non-Z-transformed interaction scores based on the area under the curve (as described in the methods section), hence raw Excess over Bliss values. This data is the direct source for figure panel 2A, and is the source for panels 2B, 2C, 2D and Supplementary Figure S2.
AUC metric z-scores: Z-transformed interaction scores based on the area under the curve (as described in the methods section). This data is used for the quality control depicted in Supplementary Figure S1.

Supplementary Data 2:
combined results: Summarized data: Enrichment values were combined across replicates by gene name. Requirement: Detection of an enrichment value in at least 2 of the 3 replicates.
R1-3 original data: Original proteomics data: SILAC enrichment ratios for all proteins detected in replicates 1-3. Additional information on number of unique peptides, protein mass and raw intensities is provided.

Supplementary Data 3:
combined results: Summarized data: Enrichment values were combined across replicates by gene name. Requirement: Detection of an enrichment value in at least 2 of the 3 replicates.
R1-3 original data: Original proteomics data: SILAC enrichment ratios for all proteins detected in replicates 1-3. Additional information on number of unique peptides, protein mass and raw intensities is provided.
