# Supplementary material for: Viral-bacterial co-infections screen in vitro reveals molecular processes affecting pathogen proliferation and host cell viability
Source: Nat Commun. 2024 Oct 4;15:8595. doi: 10.1038/s41467-024-52905-2 (PMC11452664; doi:10.1038/s41467-024-52905-2)
Supplement: Supplementary file 7 — Reporting Summary [file 41467_2024_52905_MOESM7_ESM.pdf]

nature portfolio

Corresponding author(s): Petr Broz

Last updated by author(s): Sep 6, 2024

Reporting Summary

Nature Portfolio wishes to improve the reproducibility of the work that we publish. This form provides structure for consistency and transparency in reporting. For further information on Nature Portfolio policies, see our Editorial Policies and the Editorial Policy Checklist.

Statistics

For all statistical analyses, confirm that the following items are present in the figure legend, table legend, main text, or Methods section.

n/a	Confirmed	
☐	☒	The exact sample size (*n*) for each experimental group/condition, given as a discrete number and unit of measurement
☐	☒	A statement on whether measurements were taken from distinct samples or whether the same sample was measured repeatedly
☐	☒	The statistical test(s) used AND whether they are one- or two-sided<br>*Only common tests should be described solely by name; describe more complex techniques in the Methods section.*
☐	☒	A description of all covariates tested
☐	☒	A description of any assumptions or corrections, such as tests of normality and adjustment for multiple comparisons
☐	☒	A full description of the statistical parameters including central tendency (e.g. means) or other basic estimates (e.g. regression coefficient) AND variation (e.g. standard deviation) or associated estimates of uncertainty (e.g. confidence intervals)
☐	☒	For null hypothesis testing, the test statistic (e.g. *F*, *t*, *r*) with confidence intervals, effect sizes, degrees of freedom and *P* value noted<br>*Give P values as exact values whenever suitable.*
☒	☐	For Bayesian analysis, information on the choice of priors and Markov chain Monte Carlo settings
☐	☒	For hierarchical and complex designs, identification of the appropriate level for tests and full reporting of outcomes
☐	☒	Estimates of effect sizes (e.g. Cohen's *d*, Pearson's *r*), indicating how they were calculated

Our web collection on statistics for biologists contains articles on many of the points above.

Software and code

Policy information about availability of computer code

Data collection	Plate reader data colllection: Biotek Cytation 5, serial number: 1602037, software version: 03/04/2017, software: Gen5 Image+ v.3.04<br>Proteomic data acquisition: Xcalibur software (Thermo Fisher Scientific)<br>Microscopy data collection: Zeiss Zen Blue software (version 3.8)
Data analysis	Proteomic data analysis: MaxQuant 2.1.4.0 and Perseus (version 1.6.15.0)<br>FACS data analysis: FlowJo v10.8.1<br>Microscopy image analysis and quantification: ImageJ (version 1.53t)<br>Statistical analyses: Prism (version 10.2.0)

For manuscripts utilizing custom algorithms or software that are central to the research but not yet described in published literature, software must be made available to editors and reviewers. We strongly encourage code deposition in a community repository (e.g. GitHub). See the Nature Portfolio guidelines for submitting code & software for further information.

Data

Policy information about availability of data

All manuscripts must include a data availability statement. This statement should provide the following information, where applicable:

- Accession codes, unique identifiers, or web links for publicly available datasets
- A description of any restrictions on data availability
- For clinical datasets or third party data, please ensure that the statement adheres to our policy

Source data are provided with this paper (see Source Data File), and has been made available in Mendeley Data (https://data.mendeley.com/datasets/thjzhzdpvc/1). This includes the calculation template, as well as original images. The proteomics data generated in this study have been deposited in the proteomeXchange repository (accession number: PXD050625, https://proteomecentral.proteomexchange.org/cgi/GetDataset?ID=PXD050625). All other data, research material or algorithms used for analysis are available upon request.

Research involving human participants, their data, or biological material

Policy information about studies with human participants or human data. See also policy information about sex, gender (identity/presentation), and sexual orientation and race, ethnicity and racism.

Reporting on sex and gender	N/A
Reporting on race, ethnicity, or other socially relevant groupings	N/A
Population characteristics	N/A
Recruitment	N/A
Ethics oversight	N/A

Note that full information on the approval of the study protocol must also be provided in the manuscript.

Field-specific reporting

Please select the one below that is the best fit for your research. If you are not sure, read the appropriate sections before making your selection.

☒ Life sciences ☐ Behavioural & social sciences ☐ Ecological, evolutionary & environmental sciences

For a reference copy of the document with all sections, see nature.com/documents/nr-reporting-summary-flat.pdf

Life sciences study design

All studies must disclose on these points even when the disclosure is negative.

Sample size	Since no animals or patients were subject in this study, no sample size needed to be predetermined. Throughout the study, relevant statistics were employed to ensure that the respective conclusions could be drawn from the data. As commonly done in the field, we performed several biological repeats per experiment, each including at least 3 technical replicates.
Data exclusions	No data was excluded after experimentation
Replication	Generally, experiments were performed in at least biological triplicate, using several technical replicates. For the screen, reproducibility was assessed after biological duplicate and a triplicate was performed when required (as described in the methodology section). We confirm that replication was successful
Randomization	This is not relevant to our study. For the plate reader data, plate effects were assessed beforehand and not deemed influencial, hence the same layout for infections was used throughout the screen.
Blinding	Since the relevant criteria in our experiments are objectively determined quantities or values (such as level of cell death or bacterial growth) a blinding of investigators is not necessary.

Reporting for specific materials, systems and methods

We require information from authors about some types of materials, experimental systems and methods used in many studies. Here, indicate whether each material, system or method listed is relevant to your study. If you are not sure if a list item applies to your research, read the appropriate section before selecting a response.

Materials & experimental systems

n/a	Involved in the study
☐	☒ Antibodies
☐	☒ Eukaryotic cell lines
☒	☐ Palaeontology and archaeology
☒	☐ Animals and other organisms
☒	☐ Clinical data
☒	☐ Dual use research of concern
☒	☐ Plants

Methods

n/a	Involved in the study
☒	☐ ChIP-seq
☐	☒ Flow cytometry
☒	☐ MRI-based neuroimaging

Antibodies

Antibodies used

Rabbit-anti-ASC, Adipogen, AL177, 1:1000 dilution
Donkey-anti-Rabbit-Alexa-647, Invitrogen, A32795, 1:5000 dilution
Rabbit-anti-Mprip, Thermo Scientific, PA5-54631, 1:1000 dilution
HRP-coupled anti-Tubulin antibody, Abcam, ab40742, 1:5000 dilution
HRP-coupled goat-anti-rabbit secondary antibody, Southern Biotech, 4030-05, 1:5000

Validation

For validation, appropriate negative controls were used throughout the work described in the manuscript. For antibody specificity, we refer to the manufacturers' websites, which include this information.

Eukaryotic cell lines

Policy information about cell lines and Sex and Gender in Research

Cell line source(s)

RAW264.7 macrophages (ATCC, TIB-71)
Bone-Marrow-derived macrophages (BMDMs, harvested from both male and female wildtype mice)
Immortalized BMDMs (iBMDMs, previously produced in the lab from BMDMs)

Authentication

None of the cell lines was authenticated

Mycoplasma contamination

Cells were tested for mycoplasma, and confirmed mycoplasma negative

Commonly misidentified lines
(See ICLAC register)

No commonly misidentified cell lines were used in the study

Plants

Seed stocks

N/A

Novel plant genotypes

N/A

Authentication

N/A

Flow Cytometry

Plots

Confirm that:

☒ The axis labels state the marker and fluorochrome used (e.g. CD4-FITC).

☒ The axis scales are clearly visible. Include numbers along axes only for bottom left plot of group (a 'group' is an analysis of identical markers).

☒ All plots are contour plots with outliers or pseudocolor plots.

☒ A numerical value for number of cells or percentage (with statistics) is provided.

Methodology

Sample preparation

To quantify bacterial uptake, BMDMs were seeded the day prior to viral infection in non-tissue culture treated 24-well plates

Sample preparation	(Eppendorff) and primed the next morning with IFN[illegible], at least 6h before mAdV2 infection at an MOI of 1, overnight. Bacteria were cultured overnight (at 37°C, Yersinia at 26°C) while shaking and Yersinia strains and mutants were subcultured (1:25 dilution) at 26°C for 1h and 37°C for 1h to induce the T3SS. Bacterial infection at MOI 5 were performed as described above, and the cells were harvested directly after invasion by incubating the infected cells for 15 minutes in chilled PBS at 4°C. Cells were detached and washed once in PBS, and subsequently stained with violet fixable violet live-dead dye (Thermo Scientific) as described by the manufacturer. Cells were washed once more and fixed in 4% PFA for 10 minutes. Subsequently, cells were transferred into 96-well U-bottom FACS plates (Brand) and flow cytometry was performed on a Cytoflex S (Beckman Coulter), where at least 20000 events were recorded for each condition in each replicate. Cells were gated by forward- and side-scatter (Cells), as well as side-scatter height and area (Single Cells) and by PB450-negativity (live cells). FITC-signal (virus) and ECD-signal (bacteria) were used for the quantification of uninfected, singly, and doubly infected cells, with respect to an uninfected control.
Instrument	Cytoflex S1 (Beckman Coulter)
Software	Acquisition: CytExpert 2.5<br>Data analysis: FlowJo v10.8.1
Cell population abundance	at least 20000 events were recorded for each condition in each replicate
Gating strategy	Cells were gated by forward- and side-scatter (Cells), as well as side-scatter height and area (Single Cells) and by PB450-negativity (live cells). FITC-signal (virus) and ECD-signal (bacteria) were used for the quantification of uninfected, singly, and doubly infected cells, with respect to an uninfected control.

☐ Tick this box to confirm that a figure exemplifying the gating strategy is provided in the Supplementary Information.
